# Pentraxin 3 deficiency ameliorates streptozotocin-induced pancreatic toxicity via regulating ER stress and β-cell apoptosis

**DOI:** 10.1016/j.mocell.2024.100168

**Published:** 2024-12-08

**Authors:** Suji Kim, Ae-Rang Hwang, Sun-Hee Kim, Jae Hyang Lim, Chang-Hoon Woo

**Affiliations:** 1 Department of Pharmacology, Yeungnam University College of Medicine, 170 Hyeonchung-ro, Nam-gu, Daegu 42415, Republic of Korea; 2 Division of Cardiovascular Disease Research, Department of Chronic Disease Convergence Research, Korea National Institute of Health, 197 Osongsaengmyeng2-ro, Osong-eub, Heungdeok-gu, Cheongju-si, Chungcheongbuk-do 28159, Republic of Korea; 3 Department of Microbiology, Ewha Womans University College of Medicine, 25 Magokdong-ro 2-gil, Gangseo-gu, Seoul 07804, Republic of Korea; 4 Senotherapy-based Metabolic Disease Control Research Center, Yeungnam University College of Medicine, 170 Hyeonchung-ro, Nam-gu, Daegu 42415, Republic of Korea

**Keywords:** β-cell, Diabetes mellitus, Endoplasmic reticulum stress, Pentraxin 3

## Abstract

The long pentraxin 3 (PTX3), a marker of inflammation, has been associated with cardiovascular disease, obesity, and metabolic syndrome. Recently, elevated serum PTX3 levels have been linked to type 2 diabetes in obese patients with nonalcoholic fatty liver disease. Diabetes mellitus is a metabolic syndrome characterized by hyperglycemia resulting from insufficient insulin secretion or action. However, the precise role of PTX3 in hyperglycemia remains unclear. This study aimed to investigate the physiological roles of PTX3 in vivo. The deformation of pancreatic islets was mitigated in PTX3-deficient mice treated with streptozotocin (STZ) compared to control C57BL/6J mice. In addition, PTX3 deficiency prevented STZ-induced unfolded protein responses and pancreatic β-cell death. Immunoblotting data revealed significant inhibition of inositol-requiring protein1α and C/EBP homologous protein (CHOP) protein expression in PTX3 KO mice administered tunicamycin which is a chemical endoplasmic reticulum stress inducer. Similarly, tunicamycin-induced Grp78, Grp94, ATF6, and CHOP mRNA levels were reduced in PTX3 KO mice. Moreover, recombinant PTX3–induced CHOP expression and β-cell apoptosis in primary mouse islets. These findings suggest that PTX3 plays a critical role in STZ-induced deformation of pancreatic islets via regulating endoplasmic reticulum stress and β-cell apoptosis.

## INTRODUCTION

Type 1 diabetes (T1D) is a chronic, metabolic disorder characterized by hyperglycemia resulting from the destruction of the insulin-producing β-cells in pancreatic islets (Banday et al., 2020, Eisenbarth et al., 1988). Streptozotocin (STZ)–induced pancreatic β-cell damage is commonly used to induce hyperglycemia in a rodent model of T1D. STZ selectively targets pancreatic β-cells by entering the cells through the highly expressed glucose transporter GLUT2, leading to DNA alkylation, DNA damage, and subsequent β-cell apoptosis (Lenzen, 2008). β-cells of the pancreatic islets are responsible for the synthesis and secretion of hormone insulin with a mission to maintain glucose homeostasis (Fu et al., 2013). Since pancreatic β-cells have a highly developed endoplasmic reticulum (ER) to support their high insulin secretory activity, they are particularly susceptible to ER stress (Fu et al., 2013). Thus, perturbations of ER homeostasis and unmitigated severe stress in β-cells lead to cell dysfunction and death, and consequently T1D pathogenesis (Sahin et al., 2021, Yong et al., 2021).

ER stress is initiated when protein homeostasis is disrupted with an accumulation of unfolded/misfolded proteins in the ER membrane. Activation of the unfolded protein response (UPR) is initiated and mediated by 3 ER stress sensors: inositol-requiring protein1α (IRE-1α), activating transcription factor 6 (ATF6), and PKR-like ER kinase (PERK) (Chen et al., 2023, Oyadomari and Mori, 2004, Sahin et al., 2021). Upon activation, ATF6 dissociation from binding immunoglobulin protein (BiP) leads to transport from ER to the Golgi apparatus where it is cleaved sequentially by serine 1 and serine 2 proteases to release its N-terminal cytoplasmic domain (Sano and Reed, 2013). In the Golgi, the ATF6 is cleaved and then moves to the nucleus and acts as a transcription factor to cause transcription of UPR target genes including X-box binding protein 1 (*XBP1*), DNA-damage inducible transcript 3 (*DDIT3*), and ER chaperones. The other 2 ER stress sensors, both IRE1α and PERK, are activated by dimerization/oligomerization and autophosphorylation following dissociation of BiP (Chen et al., 2023). The active IRE1 nonconventionally cleaves substrate precursor mRNA of XBP1 to mature XBP1s mRNA (spliced XBP1 mRNA) that regulates lipid and glucose metabolism by promoting ER-associated protein degradation, and also expression of ER chaperones, including BiP. The next phase of activated PERK phosphorylates eukaryotic initiation factor 2α which in turn activates the transcription factor ATF4, leading to induction of ER stress target gene DDIT3 which is known as C/EBP homologous protein (CHOP) (Oyadomari and Mori, 2004). The transcription factor CHOP is a key regulator of ER stress–induced apoptosis. Interestingly, Nam et al. (2015) showed that CHOP deficiency protects methylglyoxal–induced cardiac dysfunction via decreased ER stress and myocyte apoptosis.

Pentraxins are a family of soluble pattern recognition proteins containing a pentraxin domain with pentraxin signature of 8 amino acids (HxCxS/TWxS, where x is any amino acid) in their C-terminal domain (Daigo et al., 2014, Norata et al., 2010). Based on the length of the primary structure, the pentraxins are divided into 2 groups: short and long pentraxins (Moalli et al., 2011). In contrast to short pentraxins, such as C-reactive protein and serum amyloid P, which are produced in the liver, the long pentraxins pentraxin 3 (PTX3) is produced in many different types of cells, including inflammatory cells and epithelial cells in response to regulating of inflammation and tissue remodeling (Doni et al., 2019, Zlibut et al., 2019). In prior studies, it was demonstrated that as a regulator of inflammatory response, PTX3 was involved in the progression of cardiovascular diseases, such as angiogenesis, atherosclerosis, extracellular matrix formation, and metabolic syndrome (Doni et al., 2019; Norata et al., 2010; Zhang et al., 2022). Interestingly, PTX3-deficient mice have been reported to be attenuated from ischemic acute kidney injury, the severity of osteoarthritis, and the metabolic inflammation associated to high fat diet-induced obesity (Bonacina et al., 2019, Chen et al., 2012, Qiu et al., 2023). On the other hand, the collective findings indicate that deficiency of the PTX3 promotes cardiac ischemia-reperfusion injury, atherosclerotic plaques develop faster, and lipopolysaccharide-induced sustained inflammation in adipose tissue (Guo et al., 2020, Norata et al., 2009, Salio et al., 2008). Recent clinical studies have reported that serum PTX3 level in obese patients with prediabetes and type 2 diabetes was higher than that of healthy groups (Karamfilova et al., 2022, Trojak et al., 2019). Furthermore, it was reported that PTX3 was positively correlated with atherosclerosis markers in patients with T1D (Katakami et al., 2013). However, the role played by PTX3 in the diabetic pancreas has not been experimentally studied yet. In this study, we studied the mechanisms underlying the PTX3 deficiency-mediated antidiabetic effects of pancreatic β-cell in a mouse model of STZ-induced diabetes.

## MATERIALS AND METHODS

### Reagents and Antibodies

STZ was sourced from Sigma. Recombinant human pentraxin 3 was purchased from Abcam. Dimethyl sulfoxide was also obtained from Sigma. Antibodies were acquired from various vendors: ATF4, GADD153 (CHOP), and β-actin from Santa Cruz; IRE1α from Cell Signaling Technology; collagen type I from Millipore; collagen type III from Fitzgerald; and tubulin from Sigma.

### Western Blot Analysis

Hearts isolated from mice were frozen in liquid nitrogen and homogenized in 0.5 mL of RIPA buffer (comprising 50 mM Tris-HCl at pH 7.4, 150 mM NaCl, 1 mM EDTA, 1% Nonidet P-40, 0.1% sodium dodecyl sulfate, 1 mM dithiothreitol, 1 mM phenylmethylsulfonyl fluoride, and a 0.01 mM protease inhibitor cocktail). Lysates were incubated with gentle agitation on ice for 15 minutes, followed by centrifugation at 15,000g for 15 minutes at 4°C. Protein concentrations were measured using the Bradford protein assay. For Western blotting, proteins were separated by SDS-PAGE, transferred onto polyvinylidene fluoride membranes, and immunoblotted with the specified primary antibodies. Subsequent immunoblotting was performed using the corresponding secondary antibodies, and signals were visualized using chemiluminescence detection reagents (Millipore) according to the manufacturer’s instructions.

### Immunostaining and TUNEL Assay

Cell death was quantified using TUNEL (terminal deoxyribonucleotide transferase–mediated dUTP nick end labeling) staining, targeting in situ DNA fragmentation, with the In Situ Cell Death Detection Kit (Roche). For immunohistochemical analyses, we used 10% paraformaldehyde-fixed, paraffin-embedded pancreas tissue sections. Sections were permeabilized with 0.2% Triton X-100 for 5 minutes and blocked with 5% goat serum in PBS for 60 minutes to prevent nonspecific binding. The sections were then incubated overnight at 4°C with a rabbit anti-insulin polyclonal antibody (Santa Cruz) at a 1:500 dilution in 1% goat serum/PBS. Alexa Fluor 546–labeled secondary antibody against mouse IgG1 (Molecular Probes) at a 1:5,000 dilution was applied for 60 minutes at room temperature. After washing, TUNEL staining was performed as per the manufacturer’s instructions, and nuclei were stained with DAPI (Invitrogen) for 10 minutes. Images were captured using an immunofluorescence microscope, and apoptotic cells were quantified using Photoshop 8.0 (Adobe Systems) by calculating the percentage of apoptotic nuclei among the total nuclei in microscopic fields under 400× magnification: percent apoptosis = (apoptotic nuclei/total nuclei) × 100.

### Quantitative Real-Time RT-PCR

mRNA expression levels were determined using quantitative real-time PCR (RT-qPCR). Total RNA was extracted using TRIzol Reagent (Invitrogen), and reverse transcription was performed with TaqMan reverse transcription reagents (Applied Biosystems) following the manufacturer’s protocols. RT-qPCR was conducted using 1 µl of template cDNA and Power SYBR Green (Applied Biosystems) in an ABI PRISM 7500 unit (Applied Biosystems). Quantification was performed using the efficiency-corrected ΔΔCq method. The primers used for DNA amplification were mouse grp94 forward and reverse; mouse grp74 forward and reverse; mouse ATF6 (NM_001081304.1) forward 5′-TCGCCTTTTAGTCCGGTTCTT-3′ and reverse 5′-GGCTCCATAGGTCTGACTCC-3′; mouse CHOP (NM_007837.4) forward 5′-GCATGAAGGAGAAGGAGCAG-3′ and reverse 5′-CTTCCGGAGAGACAGACAGG-3′; and mouse GAPDH (NM_001289726) forward 5′-GGAGCCAAAAGGGTCATCAT-3′ and reverse 5′-i-3′.

### Mouse Islet Isolation

Islets were isolated from the pancreas of male C57BL/6J mice (25-30 g) injected with 3 ml of 1 mg/ml collagenase in HBSS modified with 25 mM HEPES and 0.25% (w/v) bovine serum albumin, adjusted to pH 7.4 with NaOH. The collagenase was injected into the pancreas via the common bile duct to expand the pancreas. The inflated pancreas was digested at 37°C in a water bath for 15 minutes with manual shaking every 5 minutes. The digested tissue was then washed at least 3 times with ice-cold modified HBSS. Islets were handpicked under a microscope and cultured in Roswell Park Memorial Institute 1640 medium containing 10% fetal bovine serum and 1% penicillin-streptomycin at 37°C in a CO_2_ incubator overnight before experiments.

### Animal Experiments

All animal experiments were conducted in accordance with protocols approved by the Institutional Animal Care and Use Committees of the College of Medicine, Yeungnam University (Daegu, Republic of Korea; Institutional Animal Care and Use Committee approval number: YUMC-AEC2015-030). PTX3-deficient mice (PTX3 KO) were provided by Dr. Martin M. Matzuk at Baylor College of Medicine under a Material Transfer Agreement. Offspring of PTX3 KO mice were backcrossed 5 times to C57BL/6 mice, which served as controls for PTX3 KO experiments. Male C57BL/6J (WT, 8 weeks old) and male PTX3 KO mice (C57BL/6J background, 8 weeks old) were injected intraperitoneally with 50 mg/kg body weight STZ. Blood glucose levels were monitored daily for 2 weeks, and mice were fasted for 12 hours with free access to water. Blood samples were collected by tail tip puncture using a glucometer (Accu-Chek; Roche). Total RNA was extracted from lung tissues, and mRNA expression of grp94, grp78, ATF6, and CHOP was measured by RT-qPCR as previously described. Pancreas tissues were fixed in 10% buffered formaldehyde and paraffin-embedded. Sections (5 µm thick) were stained with H&E.

### Statistical Analysis

Data are presented as means ± SD, and the significances of intergroup differences were assessed by unpaired Student’s *t*-test and multiple group comparisons using ANOVA followed by Bonferroni’s post hoc test, with *P*-values <.05 indicative of a significant difference. The analysis was conducted using GraphPad Prism (Graph-Pad Software Inc.).

## RESULTS

### Deficiency of PTX3 Ameliorated STZ-Induced Pancreatic Toxicity

**Fig. 1:**
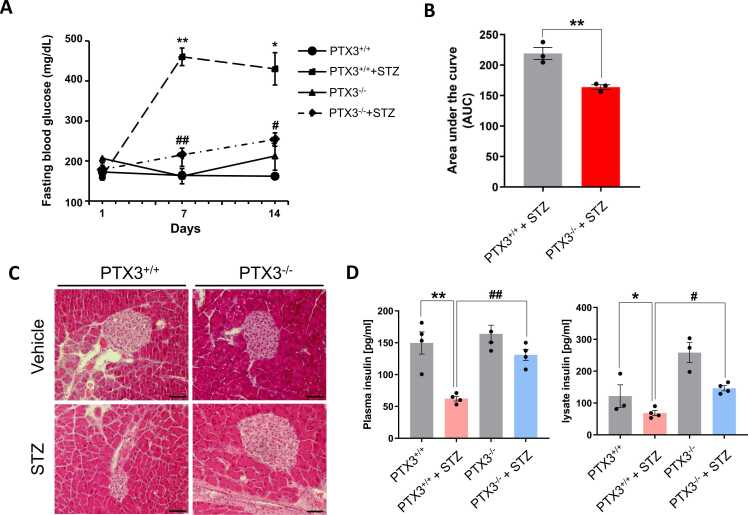
PTX3 deficiency inhibits STZ–induced pancreatic injury. Mice were injected intraperitoneally with low-dose streptozotocin (STZ, 50 mg/kg) for 5 consecutive days. (A) Change in blood glucose. Results are presented as means ± SDs (*n* = 6 per group). ANOVA: *P < .05 PTX3 WT vs PTX3 WT-STZ; **P < .01 PTX3 WT vs PTX3 WT-STZ; ^#^*P* < .05 STZ vs PTX3 KO-STZ; ^##^*P* < .01 STZ vs PTX3 KO-STZ. (B) Area under the curve (AUC) of fasting blood glucose. *t*-test: **P < .01. (C) Histology of mouse pancreas stained with hematoxylin and eosin. The pancreatic islets are brighter than the pancreatic tissue. Images were observed under a fluorescence microscope (original magnification, 200×, scale bars: 50 µm). (D) ELISA for insulin levels in plasma and pancreatic tissues (*n* = 3 per group). ANOVA: *P < .05 PTX3 WT vs PTX3 WT-STZ; **P < .01 PTX3 WT vs PTX3 WT-STZ; ^#^*P* < .05 STZ vs PTX3 KO-STZ; ^##^*P* < .01 STZ vs PTX3 KO-STZ.

Recent reports suggest that PTX3 was implicated as a new diagnostic biomarker of vascular disease in type 2 diabetes mellitus and diabetic cardiac dysfunction after myocardial infarction (Salio et al., 2008, Trojak et al., 2019). To determine the role of PTX3 in the pathogenesis of hyperglycemia, we first examined if PTX3 affects hyperglycemia and pancreatic β-cell death in an STZ–induced mouse model of T1D. Control C57BL/6 mice and PTX3 KO were inoculated intraperitoneally with STZ. Fasting blood glucose levels were increased in STZ in C57BL/6 mice, and it was significantly decreased in STZ-treated PTX3 KO (Fig. 1A and B). In addition, histological analyses were performed on pancreas tissue section from STZ-induced C57BL/6 mice and PTX3 KO mice. H&E staining of pancreas sections showed the destruction of pancreatic islet in C57BL/6 mice, whereas STZ infusion PTX3 KO mice were inhibited in deformation of pancreatic islets (Fig. 1C). In addition, we found that STZ-induced reduction of insulin in plasma and pancreatic tissues was reversed in PTX3 KO (Fig. 1D). Taken together, these data indicate that deficiency of PTX3 suppresses ameliorates STZ-induced pancreatic islet damage, ultimately reducing hyperglycemia.

### PTX3 Deficiency Ameliorated STZ-Induced Apoptosis and ER Stress of Mouse Islets

**Fig. 2:**
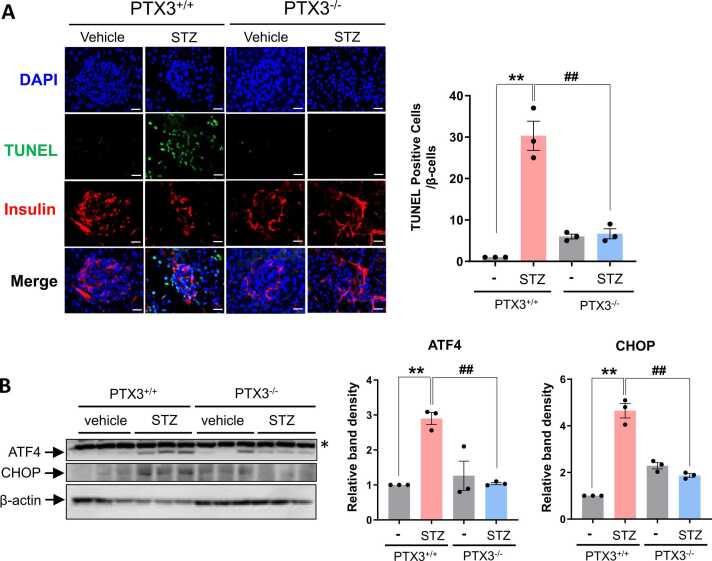
PTX3 deficiency ameliorated STZ-induced apoptosis and ER stress of mouse islets. Mice received a single *i.p.* injection of STZ (50 mg/kg) whereas vehicles were administered citrate buffer. (A) The pancreatic sections were subjected to TUNEL staining. Representative photomicrographs showing TUNEL (apoptotic, green), insulin (pancreatic β-cells, red), and Topro 3 (nuclei, blue) signals and merged images (original magnification, 400×, scale bars: 50 µm). Bar graphs present percentages of numbers of TUNEL-positive cells among total numbers of pancreatic β-cells counted (*n* = 3 per group). ANOVA: **P < .01 PTX3 WT vs PTX3 WT-STZ; ^##^*P* < .01 STZ vs PTX3 KO-STZ. (B) Protein levels were measured by immunoblotting with antibodies against ATF4, CHOP, and β-actin. Results are expressed as mean ± SD values as bar graph (*n* = 3 per group). ANOVA: **P < .01 PTX3 WT vs PTX3 WT-STZ; ^##^*P* < .01 STZ vs PTX3 KO-STZ. Asterisks indicate nonspecific bands.

Having demonstrated that PTX3 regulates STZ–induced β-cell destruction, we next investigated if PTX3 expression increases β-cell apoptosis induced by STZ. To visualize the deformation of pancreas, we performed the immunostaining with anti-insulin antibody. Immunostaining data showed that the size of islets challenged with STZ was smaller and that there were more TUNEL-positive cells in wild-type mice compared to PTX3 KO, suggesting that PTX3 KO inhibits STZ induced the deformation of pancreatic islets and β-cells death (Fig. 2A).

It has been established that ER stress contributes to the pathogenesis of diabetes and β-cell apoptosis (Yong et al., 2021), and thus we evaluated UPRs in pancreas tissues from STZ infusion mice. As shown in Figure 2B, protein expression levels of ATF4 and CHOP were significantly increased in STZ–induced C57BL/6 mice, and that these changes were decreased in STZ–treated PTX3-deficient mice. These results suggest that the protective effect of PTX deficiency on STZ–induced pancreatic β-cell apoptosis and ER stress.

### PTX3 Deficiency Ameliorates Tunicamycin-Induced UPR in Pancreas

**Fig. 3:**
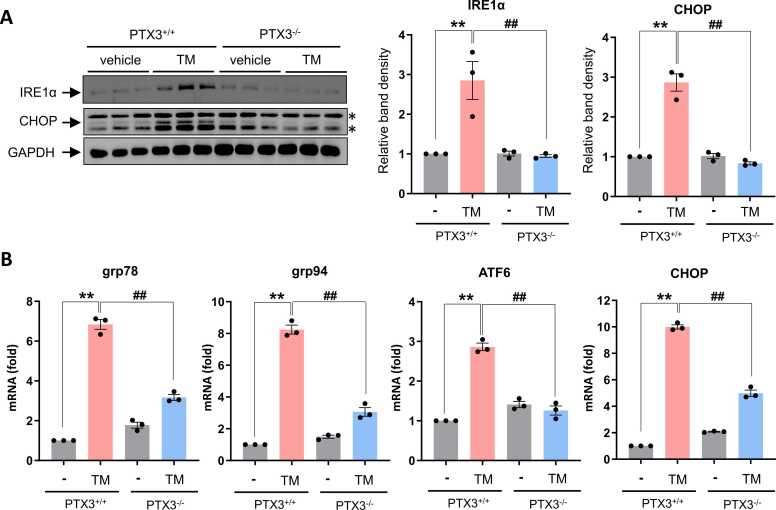
PTX3 deficiency ameliorates tunicamycin-induced UPR in pancreas. PTX3 WT or PTX3 KO mice were injected with tunicamycin (*i.p.* 2 mg/kg) for 3 days, and the pancreas tissues were harvested. (A) Protein levels were measured by immunoblotting with antibodies against IRE1α, CHOP, and β-actin. Results are expressed as mean ± SD values as bar graph (*n* = 3 per group). ANOVA: **P < .01 PTX3 WT vs PTX3 WT-STZ; ^##^*P* < .01 STZ vs PTX3 KO-STZ. Asterisks indicate nonspecific bands. (B) mRNA levels of grp94, grp78, ATF6, and CHOP were measured by performing RT-qPCR. Relative expression levels were normalized to GAPDH. Results are expressed as mean ± SD values as bar graph (*n* = 3 per group). ANOVA: **P < .01 PTX3 WT vs PTX3 WT-STZ; ^##^*P* < .01 STZ vs PTX3 KO-STZ.

STZ-induced hyperglycemia has been reported to accelerate ER stress–mediated pancreatic β-cell apoptosis in a murine model (Ahn et al., 2015). To examine if PTX3 is involved in TM–induced ER stress in vivo, control mice and PTX3 KO mice were subjected to TM (2 µg/kg body weight, *i.p.*) for 36 hours. Immunoblotting data showed that protein expression of IRE1α and CHOP were significantly inhibited in TM–induced PTX3 KO mice compared to control mice (Fig. 3A). Similarly, TM-induced grp78, 94, ATF6, and CHOP mRNA levels were inhibited in PTX3 KO mice (Fig. 3B). These observations suggested that PTX3 deficiency inhibits TM-induced UPRs in pancreas.

### Recombinant PTX3-Induced CHOP and Apoptosis in Mouse Islets

**Fig. 4:**
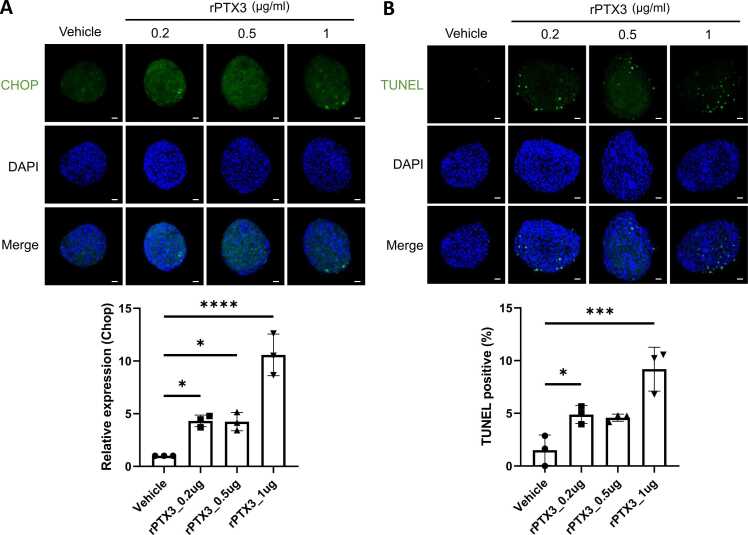
Recombinant PTX3-induced CHOP and apoptosis in mouse islets. Pancreatic islets were treated with recombinant PTX3 (0.2-1 µg/ml) for overnight and then immunostained for CHOP and TUNEL staining. (A) Immunofluorescence image of CHOP (green), DAPI (blue), and merged images (original magnification, 200×, scale bars: 30 µm) in mouse islets (*n* = 3 per group). ANOVA: **P < .05; ***P < .001 vs vehicle. (B) Representative photomicrographs showing the pancreatic islets labeled with TUNEL (apoptotic, green), DAPI (nuclei, blue) signals, and their merged images (original magnification, 200×, scale bars: 30 µm) (*n* = 3 per group). ANOVA: **P < .05; ***P < .001 vs vehicle.

Since we have shown that PTX3 deficiency blocks STZ-induced UPRs and β-cell apoptosis in vivo, we evaluated if PTX3 regulates CHOP and β-cell apoptosis. To better understand the role of PTX3, we utilized a primary islet-based cell culture system. As shown in Figure 4A, recombinant PTX3 markedly increased CHOP expression in primary mouse islets. Next, TUNEL-positive cells in primary mouse islets were dramatically increased in a dose-dependent manner by recombinant PTX3 (Fig. 4B). These results suggest that recombinant PTX3 regulates CHOP and apoptosis in primary mouse islets.

## DISCUSSION

The findings presented in this study shed light on the pivotal role of PTX3 in the pathogenesis of hyperglycemia, pancreatic ER stress, and β-cell apoptosis. The study employed a combination of in vivo experiments using STZ–induced diabetic mouse models and in vitro analyses utilizing primary mouse islets. These results demonstrate that PTX3 deficiency exerts a protective effect against STZ-induced deformation of pancreatic islets and β-cell death, highlighting PTX3 as a potential therapeutic target for diabetes mellitus.

The observed mitigation of hyperglycemia in PTX3-deficient mice treated with STZ suggests a critical involvement of PTX3 in the regulation of glucose homeostasis. This finding aligns with previous reports linking elevated serum PTX3 levels to type 2 diabetes in obese patients with nonalcoholic fatty liver disease, indicating the clinical relevance of PTX3 in metabolic disorders (Karamfilova et al., 2022, Trojak et al., 2019). Furthermore, histological analyses revealed that PTX3 deficiency attenuated the deformation of pancreatic islets induced by STZ, providing additional evidence for the protective role of PTX3 depletion against β-cell damage (Figs. 1 and 2).

Intriguingly, the study also elucidated the mechanistic underpinnings of PTX3-mediated effects on pancreatic ER stress and β-cell apoptosis. Immunoblotting data demonstrated that PTX3 deficiency inhibited the expression of key ER stress markers, including IRE1α and CHOP, in TM-treated mice (Fig. 3). Since TM is an ER stress inducer acting via inhibition of protein N-linked glycosylation and persistent ER stress could trigger apoptotic cell death, we utilized TM as inducer for ER stress–dependent cell death. Similarly, TM-induced UPRs were attenuated in PTX3 knockout mice, suggesting that PTX3 plays a critical role in ER stress modulation. These findings are particularly significant considering the established association between ER stress and diabetes mellitus, underscoring PTX3 as a potential regulator of ER stress–mediated β-cell dysfunction and apoptosis.

Moreover, the study elucidated the direct effect of PTX3 on β-cell apoptosis using primary mouse islets. Recombinant PTX3 treatment led to increased expression of CHOP, a key mediator of ER stress–induced apoptosis, and elevated apoptosis in a dose-dependent manner (Fig. 4). These results provide mechanistic insights into the proapoptotic role of PTX3 in pancreatic islets, further supporting its involvement in β-cell demise. The molecular mechanism by which PTX3 induces cell death remains uncertain. Studies have shown that PTX3 plays a role in modulating the inflammatory response and the UPR, suggesting that PTX3 deficiency may alter UPR signaling pathways under stress conditions (Deban et al., 2011). In addition, Carrizzo et al. (2019) reported that rPTX3 induces cytoplasmic vacuolization and mitochondrial matrix dilution in endothelial cells. These findings suggest that PTX3-mediated inflammation and mitochondrial dysfunction may be involved in the cell death pathway.

The implications of these findings extend beyond our understanding of PTX3's role in diabetes mellitus to its potential as a therapeutic target. Given the observed protective effects of PTX3 deficiency against STZ-induced deformation of pancreatic islets and β-cell death, targeting PTX3 may represent a promising strategy for mitigating diabetes-related complications. Future studies investigating the molecular mechanisms underlying PTX3-mediated effects and exploring therapeutic interventions targeting PTX3 are warranted to harness its therapeutic potential fully.

In conclusion, this study provides compelling evidence implicating PTX3 in the pathogenesis of hyperglycemia, pancreatic ER stress, and β-cell apoptosis. The findings underscore the significance of PTX3 as a potential therapeutic target for diabetes mellitus and highlight the need for further research to elucidate its precise mechanisms of action and therapeutic implications.

## Author Contributions

Conceptualization: Suji Kim, Ae-Rang Hwang, Jae Hyang Lim, and Chang-Hoon Woo. Investigation: Suji Kim, Ae-Rang Hwang, and Sun-Hee Kim. Data curation: Suji Kim and Chang-Hoon Woo, Writing—original draft preparation: Suji Kim and Sun-Hee Kim. Writing—review and editing: Suji Kim, Ae-Rang Hwang, Jae Hyang Lim, and Chang-Hoon Woo. Supervision: Jae Hyang Lim and Chang-Hoon Woo. All authors have read and agreed to the published version of the manuscript.

## Declaration of Competing Interests

The authors declare that they have no known competing financial interests or personal relationships that could have appeared to influence the work reported in this paper.
