# Supplementary material for: Pentraxin 3 deficiency ameliorates streptozotocin-induced pancreatic toxicity via regulating ER stress and β-cell apoptosis
Source: Mol Cells. 2024 Dec 8;48(1):100168. doi: 10.1016/j.mocell.2024.100168 (PMC11742826; doi:10.1016/j.mocell.2024.100168)
Supplement: Supplementary file 1 — Supplementary material [file mmc1.pptx]

## Slide 1
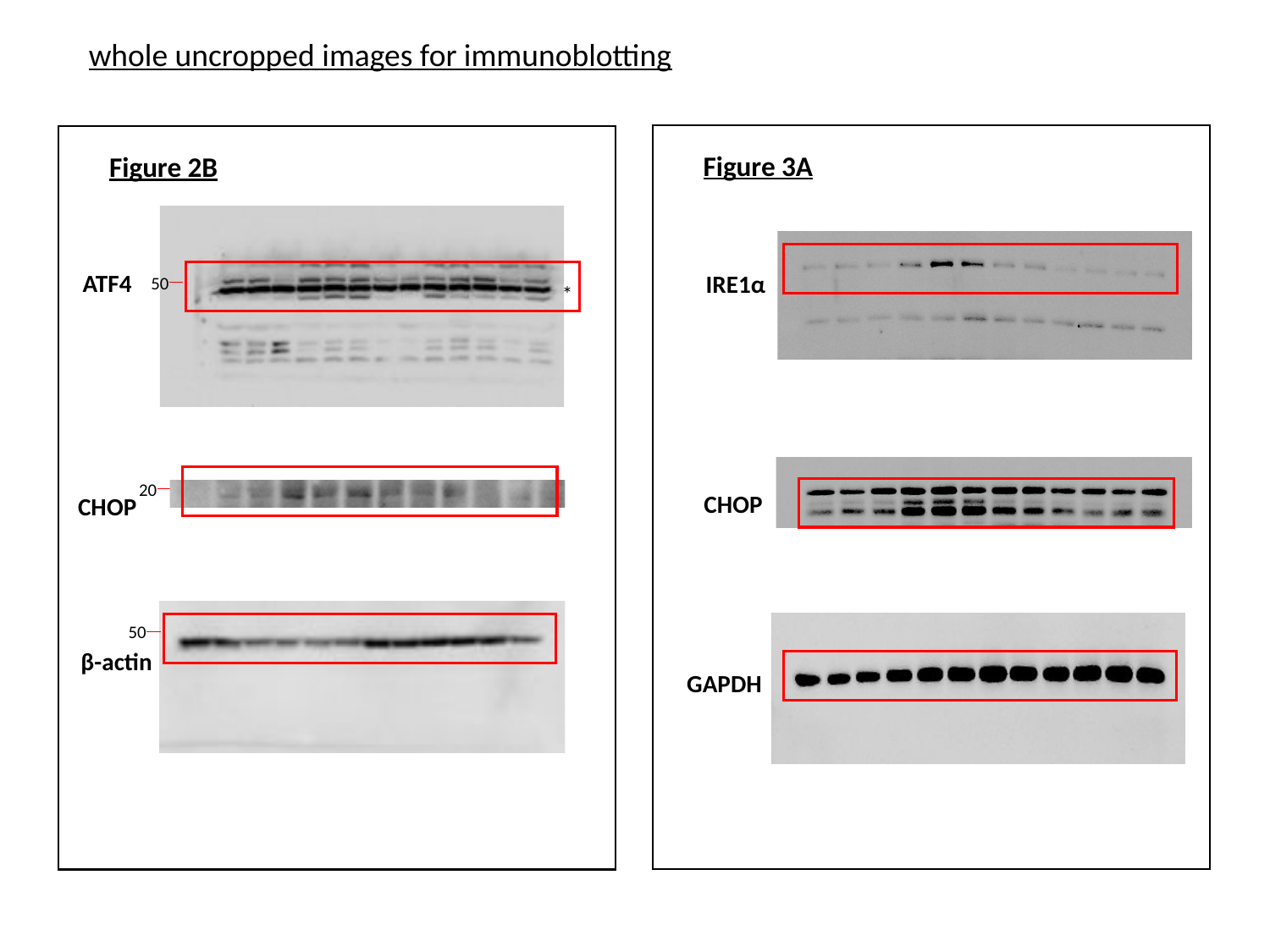

whole uncropped images for immunoblotting
Figure 3A
Figure 2B
ATF4
IRE1α
50
*
20
CHOP
CHOP
50
β-actin
GAPDH
